# Expression of signal-transducing adaptor protein-1 attenuates experimental autoimmune hepatitis via down-regulating activation and homeostasis of invariant natural killer T cells

**DOI:** 10.1371/journal.pone.0241440

**Published:** 2020-11-11

**Authors:** Jun-ichi Kashiwakura, Kodai Saitoh, Takeru Ihara, Yuto Sasaki, Kota Kagohashi, Shiyo Enohara, Yuka Morioka, Hiroshi Watarai, Ryuta Muromoto, Yuichi Kitai, Kazuya Iwabuchi, Kenji Oritani, Tadashi Matsuda

**Affiliations:** 1 Department of Immunology, Graduate School of Pharmaceutical Sciences, Hokkaido University, Sapporo, Hokkaido, Japan; 2 Division of Disease Model Innovation, Institute for Genetic Medicine, Hokkaido University, Sapporo, Hokkaido, Japan; 3 Department of Immunology and Stem Cell Biology, Faculty of Medicine, Institute of Medical, Pharmaceutical and Health Sciences, Kanazawa University, Kanazawa, Ishikawa, Japan; 4 Department of Immunology, Kitasato University Graduate of School of Medical Science, Sagamihara, Kanagawa, Japan; 5 Department of Hematology, International University of Health and Welfare, Narita, Chiba, Japan; Institut d'Investigacions Biomediques de Barcelona, SPAIN

## Abstract

**Objective:**

Signal-transducing adaptor protein (STAP) family members function as adaptor molecules and are involved in several events during immune responses. Notably however, the biological functions of STAP-1 in other cells are not known. We aimed to investigate the functions of STAP-1 in invariant natural killer T (iNKT) cells and iNKT cell-dependent hepatitis.

**Methods:**

We employed concanavalin A (Con A)-induced hepatitis and α-galactosylceramide (α-GalCer)-induced hepatitis mouse models, both are models of iNKT cell-dependent autoimmune hepatitis, and STAP-1 overexpressing 2E10 cells to investigate the role of STAP-1 in iNKT cell activation in vivo an in vitro, respectively.

**Results:**

After Con A- or α-GalCer-injection, hepatocyte necrotic areas and plasma alanine aminotransferase elevation were more severe in STAP-1 knockout (S1KO) mice and milder in lymphocyte-specific STAP-1 transgenic (S1Tg) mice, as compared to wild-type (WT) mice. Two events that may be related to Con A-induced and/or α-GalCer-induced hepatitis were influenced by STAP-1 manipulation. One is that iNKT cell populations in the livers and spleens were increased in S1KO mice and were decreased in S1Tg mice. The other is that Con A-induced interleukin-4 and interferon-γ production was attenuated by STAP-1 overexpression. These effects of STAP-1 were confirmed using 2E10 cells overexpressing STAP-1 that showed impairment of interleukin-4 and interferon-γ production as well as phosphorylation of Akt and mitogen-activated protein kinases in response to Con A stimulation.

**Conclusions:**

These results conclude that STAP-1 regulates iNKT cell maintenance/activation, and is involved in the pathogenesis of autoimmune hepatitis.

## Introduction

Autoimmune hepatitis is an inflammatory immune disease of the liver, and a worldwide health problem in humans. Because the only efficient therapeutic medication is glucocorticoid, patient quality of life is not high [1, 2]. A better understanding of the mechanisms involved in autoimmune hepatitis is needed to facilitate the development of new therapeutic medicines. Concanavalin A (Con A)-induced liver injury in mice is phenotypically similar to autoimmune hepatitis [3–5]. Notably, murine Con A-induced hepatitis is evidently dependent on T cells, because liver injury after the administration of Con A is attenuated in both T cell-deficient athymic nude mice and severe combined immunodeficiency mice [3].

Invariant natural killer T (iNKT) cells are innate-like T lymphocytes that express an invariant T cell antigen receptor encoded by Vα14Jα18 gene segments [6]. iNKT cells recognize a synthetic glycolipid, α-galactosylceramide (α-GalCer), and bacterial glycosphingolipids such as α-linked glucuronic acid. Upon stimulation with α-GalCer, iNKT cells secrete interleukin-4 (IL-4) and interferon-γ (IFN-γ) [7]. Two recent studies suggest the importance of iNKT cells and iNKT cell-derived IL-4 in the pathogenesis of Con A-induced hepatitis. Toyabe et al. reported that natural killer (NK)1.1^+^ cells are crucial for the development of Con A-induced hepatitis [8]. Kaneko et al. reported that *Traj18*-deficient iNKT-lacking mice don’t exhibit liver injury after Con A treatment [9]. In the latter report, requirement of iNKT cell-derived IL-4 for Con A-induced hepatitis was also demonstrated. Suppressor of cytokine signaling 3 (SOCS 3) in iNKT cells was also reported to be involved in the pathogenesis of Con A-induced hepatitis [10]. Tiegs and colleagues demonstrated that TNF-α is an important cytokine for α-GalCer-induced liver injury in mice [11]. IL-12, a type I helper T cell cytokine, is involved in pathogenesis of Con A-induced hepatitis by enhancing IL-4 production in iNKT cells [12]. However, the precise molecular mechanisms are currently unknown.

Signal-transducing adaptor protein (STAP) family members function as adaptor molecules and are involved in several events during immune responses. We have previously investigated STAP-2, which is involved in various immune signaling events such as FcεRI-mediated mast cell/basophil activation [13, 14], integrin/chemokine receptor-mediated T cell adhesion/migration [15, 16], and Toll-like receptor-mediated monocyte activation [17].

STAP-1 is a Tec tyrosine kinase binding partner that is predominantly expressed in the spleen and lymph nodes [18]. Mouse STAP-1 has been cloned as a c-Kit-binding protein using a mouse hematopoietic stem cell library [19]. STAP-1 contains a pleckstrin homology domain in its N terminal half and a Src homology 2 domain in its C terminal half, and is believed to act as an adaptor protein. It has been reported that in structural biological investigations the STAP-1 Src homology 2 domain could bind with non-T cell activation linker via its P+4 binding pocket [20]. There are three isoforms of mouse STAP-1, and only the longest one has the capacity to interact with Tec [21]. STAP-1 has been reported to be an important molecule in the regulation of chemotaxis and phagocytosis in microglia [22]. Gene mutations in *STAP1* was identified in patients with autosomal dominant hypercholesterolemia [23, 24] although the role of STAP-1 in cholesterol homeostasis is still controversial [25, 26].

Although several reports have suggested some functions of STAP-1, it is unknown whether STAP-1 is involved in the pathogenesis of immune diseases such as autoimmunity and allergy. In the present study we demonstrated that STAP-1 is required for the maintenance/activation of iNKT cells, and has a capacity to modify autoimmune hepatitis.

## Materials and methods

### Antibodies

FITC-anti-mouse TCRβ (clone: H57-597), PerCP/Cy5.5-anti-mouse/human CD44 (clone: IM7), PE/Dazzle^TM^ 594-anti-mouse CD24 (clone: M1/69) and PE-anti-mouse NK1.1 (clone: PK136) mAbs were purchased from BioLegend (San Diego, CA, USA). An anti-STAP-1 mAb (clone: S1/1) was generated in mice by immunization with recombinant STAP-1 as previously described [27].

### Mice

C57BL/6N mice were purchased from SANKYO LABO SERVICE CO. Inc. (Hokkaido, Japan). A C57BL/6N background STAP-1 KO ES cells (EPD0583_5_G02) were purchased from European Conditional Mouse Mutagenesis Program. Human STAP-1 cDNA was inserted into the p1026x vector that consists of the murine lck proximal promoter, Ig intronic H chain enhancer Eμ, and a human growth hormone (hGH) gene cassette [28]. The Stap1 transgene fragment was injected into C57BL/6 mouse zygote pronuclei, and transgenic mice were generated. All animal studies were approved by the Hokkaido University animal ethics committee (Approval number: 18–0024). All mice were housed and bred in the Pharmaceutical Sciences Animal Center of Hokkaido University under specific pathogen-free conditions.

### Hepatitis mouse models

The mice were intravenously injected with Con A (10 mg/kg, Sigma-Aldrich, St Louis, MO, USA) or α-GalCer (0.1 mg/kg, Funakoshi, Tokyo, Japan) [10]. Plasma ALT levels were measured using SRL service. IL-4 and IFN-γ levels were measured using ELISA kits (BioLegend). Formalin-fixed paraffin-embedded liver sample specimens (5 μm) were stained with hematoxylin and eosin. Necrotic areas in the livers were measured using ImageJ program (NIH, Bethesda, MD, USA)

### Flowcytometric analysis

Flowcytometric analysis was performed as previously described [14]. Fluorescence of the stained cells was detected using Gallios (BECKMAN COULTER, Inc. Brea, CA, USA) and analyzed using FlowJo software version 10 (FlowJo, LLC, Ashland, OR, USA).

### Establishment of STAP-1 overexpressing 2E10 cells

Murine iNKT cell hybridoma, 2E10 [29], is cultured in 10% FCS RPMI1640. For establishment of STAP-1 overexpressing 2E10 cells, 2E10 cells were first transfected with 20 μg of 6xMyc-tagged STAP-1-expressing pcDNA3.1(+) vector by electroporation. After drug resistant 2E10 cells were generated, the cells were cloned by a limiting dilution method. After cloning, 6xMyc-tagged STAP-1 was detected by western blot analysis and generated clones were used for the experiments.

### Western blot analysis

Western blot analysis was performed as previously described [13]. NIH-3T3 and Ba/F3 cell lines were purchased from American Type Culture Collection (Manassas, VA, USA)

### Statistical analysis

Statistical analysis was performed using GraphPad Prism 6.02. Mann-Whitney U-test was employed. Data were considered significant at p<0.05. Data were shown mean + SEM.

## Results

### Expression of STAP-1

**Fig 1:**
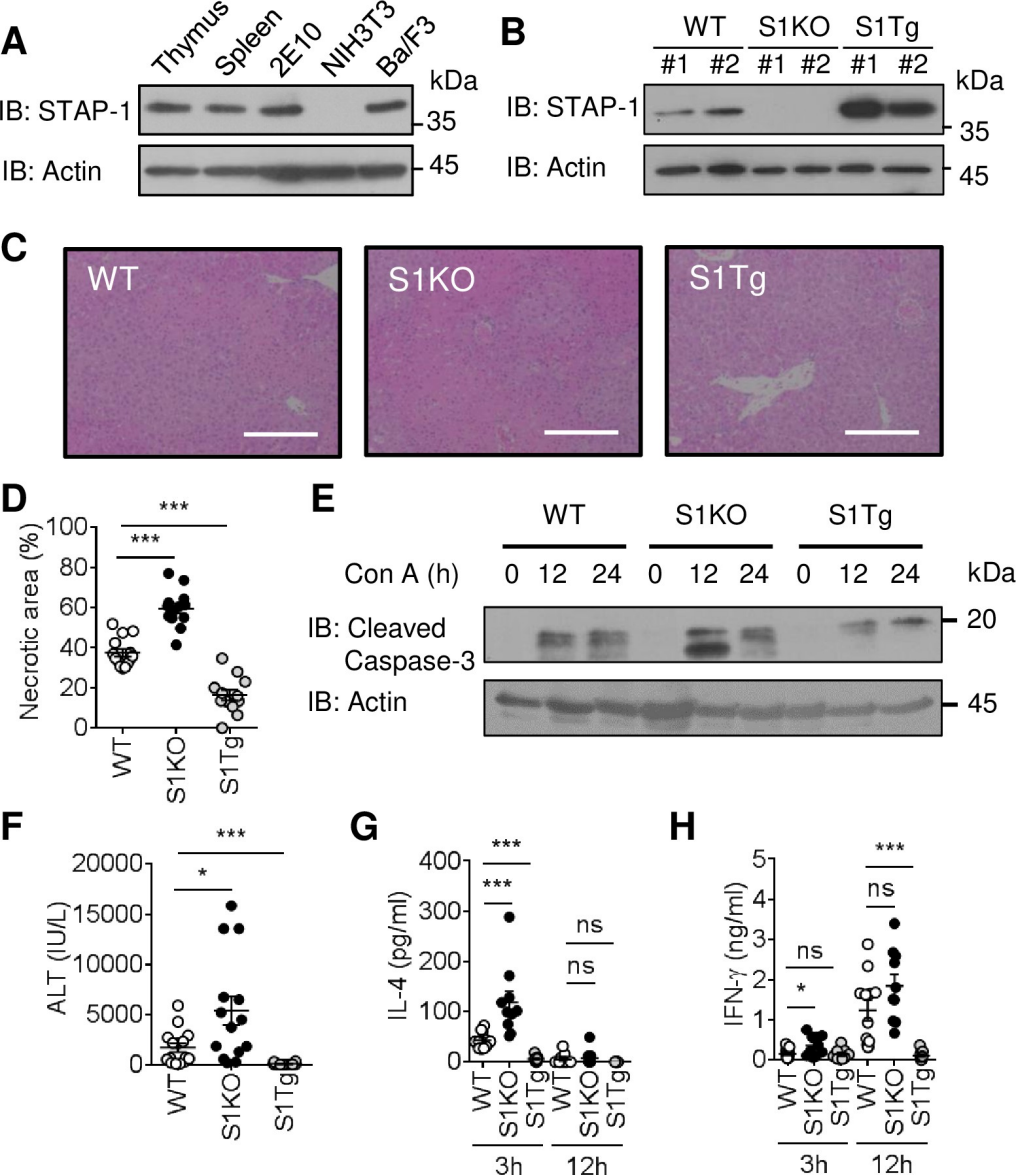
Con A-induced hepatitis in STAP-1 KO and Tg mice. **(A)** STAP-1 expression in thymus and spleen from C57BL/6 mouse, 2E10 (mouse NKT cells), NIH-3T3 (mouse fibroblasts) and Ba/F3 (pro-B cells) was detected by western blotting. Actin was detected as a loading control. **(B)** STAP-1 expression in spleens of C57BL/6 (WT), STAP-1 KO (S1KO) and STAP-1 Tg (S1Tg) mice was detected by western blotting. Actin was detected as a loading control. **(C)** WT, S1KO and S1Tg mice were intravenously inject with Con A (10 mg/kg). After Con A injection, mice were sacrificed, and liver tissues were collected. The necrotic areas in livers of WT, S1KO and S1Tg mice at 12 h after intravenous injection of Con A was visualized by H&E staining. Representative images of H&E staining from four independent experiments with a total 12–14 mice are shown. Magnification; Objective x 4, Optical x 12, Scale bar = 200 μm. **(D)** Necrotic area percentages in livers of WT, S1KO and S1Tg mice at 12 h after intravenous injection of Con A were measured by ImageJ. Four independent experiments were performed and pooled data were shown mean + SEM (WT n = 14, S1KO n = 13, S1Tg n = 12). **(E)** Liver tissues were collected from WT, S1KO and S1Tg mice before and after intravenous Con A injection (12 or 24 h), cleaved caspase-3 in the livers was detected by western blotting. Actin was detected as a loading control. **(F)** Plasma ALT levels of WT, S1KO and S1Tg mice at 12 h after intravenous injection of Con A were measured. Four independent experiments were performed and pooled data were shown mean + SEM (WT n = 15, S1KO n = 14, S1Tg n = 12). (G, H) Plasma IL-4 and IFN-γ levels of WT, S1KO and S1Tg mice at 3 and 12 h after intravenous injection of Con A were measured by ELISA. Three independent experiments were performed and pooled data were shown mean + SEM (WT n = 11, S1KO n = 10, S1Tg n = 9). *; p<0.05, ***; p<0.001 by Mann-Whitney U-test. ns = no significance.

Endogenous STAP-1 expression was tested in splenic and thymic tissues from C57BL/6 mice, and in cell lines including NIH-3T3 (fibroblasts), Ba/F3 (pro B cells), and 2E10 (iNKT cells). It was detected in the murine splenic and thymic tissues and in Ba/F3 cells and 2E10 cells, but not in NIH-3T3 cells (Fig 1A). To investigate STAP-1 expression levels, STAP-1 protein was assessed in wild-type (WT), STAP-1 KO (S1KO), and lymphocyte-specific STAP-1 transgenic (S1Tg) mice. STAP-1 protein expression in the spleen was absent in STAP-1 KO (S1KO) mice but abundant in lymphocyte-specific STAP-1 transgenic (S1Tg) mice (Fig 1B).

### Role of STAP-1 for Con A- or α-GalCer-induced hepatitis

To investigate whether STAP-1 is involved in the pathogenesis of autoimmune hepatitis, Con A-induced hepatitis—a murine model of autoimmune hepatitis—was induced in S1KO, S1Tg, and WT mice. Liver tissues from Con A-treated S1KO mice exhibited more severe necrosis than those from WT mice, whereas Con A-treated S1Tg mice exhibited less liver injury than WT mice (Fig 1C). The differences were confirmed by measuring necrotic areas in livers of each mouse strain after Con A injection. The necrotic areas in S1KO mice were significantly largest, and those in S1Tg mice were smallest (Fig 1D). Compared to WT mice, cleaved caspase-3 levels were increased in Con A-treated S1KO mice but reduced in Con A-treated S1Tg mice (Fig 1E). Plasma alanine aminotransferase (ALT) levels were also measured. Compared to WT mice, plasma alanine aminotransferase (ALT) levels were significantly higher in Con A-treated S1KO mice but lower in Con A-treated S1Tg mice (Fig 1F). These results suggest that STAP-1 acts as a negative regulator of Con A-induced hepatitis.

IL-4 and IFN-γ are important cytokines to mediate murine Con A-induced hepatitis [9, 30], thus plasma levels of each factor were measured in Con A-treated WT, S1KO, and S1Tg mice. Compared to WT mice, significantly increased plasma IL-4 (3 h) and IFN-γ (3 h) were observed in S1KO mice, and both plasma IL-4 (3 h) and plasma IFN-γ (12 h) were significantly reduced in S1Tg mice (Fig 1G and 1H). These results suggest that STAP-1 negatively regulates murine Con A-induced hepatitis via impaired IL-4 and/or IFN-γ production.

**Fig 2:**
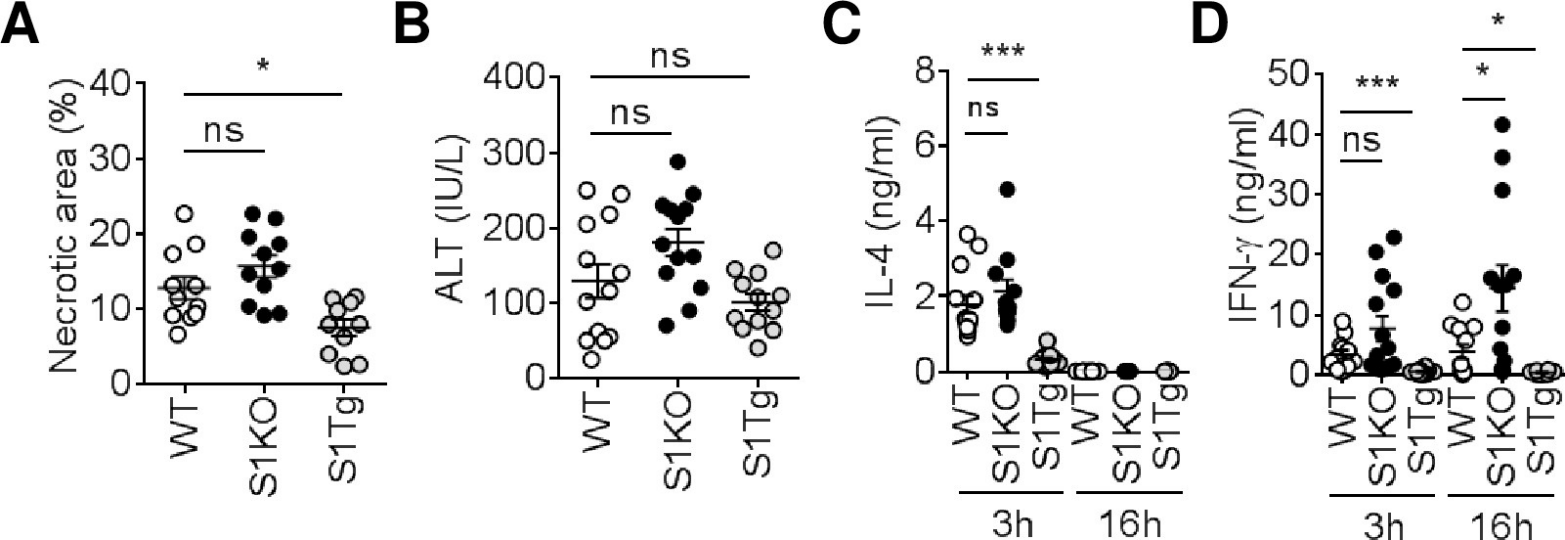
α-GalCer-induced hepatitis in STAP-1 KO and Tg mice. **(A)** The necrotic areas in livers of C57BL/6 (WT), S1KO and S1Tg mice at 16 h after intravenous injection of α-GalCer (0.1 mg/kg) was visualized by H&E staining and percentages of the area were measured by ImageJ. Three independent experiments were performed and pooled data were shown mean + SEM (WT n = 11, S1KO n = 11, S1Tg n = 10). **(B)** Plasma ALT levels of WT, S1KO and S1Tg mice at 16 h after intravenous injection of α-GalCer were measured. Three independent experiments were performed and pooled data were shown mean + SEM (WT n = 14, S1KO n = 14, S1Tg n = 12). **(C, D)** Plasma IL-4 and IFN-γ levels of WT, S1KO and S1Tg mice at 3 and 16 h after intravenous injection of α-GalCer were measured by ELISA. Three independent experiments were performed and pooled data were shown mean + SEM (WT n = 14, S1KO n = 13–14, S1Tg n = 11–13). *; p<0.05, ***; p<0.001 by Mann-Whitney U-test. ns = no significance.

α-GalCer is a stimulator, which is more restricted to iNKT than Con A. To investigate whether STAP-1 is a critical adaptor protein to induce iNKT cell-mediated hepatitis, the effects of STAP-1 on α-GalCer-induced hepatitis were assessed [10]. To confirm the effects of STAP-1 on iNKT cell-mediated hepatitis, we measured necrotic areas of livers in α-GalCer-treated WT, S1KO, and S1Tg mice. Compared to WT livers, necrotic areas were slightly increased in S1KO livers but significantly reduced in S1Tg livers (Fig 2A). Compared to the WT mice, plasma ALT levels were increased in S1KO mice and decreased in S1Tg mice, but neither of these differences were statistically significant (Fig 2B). Lastly, plasma IL-4 and IFN-γ levels were measured. Compared to WT mice, in S1KO mice IL-4 levels tended to be higher 3 h after α-GalCer administration, and IFN-γ levels were significantly higher 16 h after α-GalCer administration. Conversely, plasma IL-4 (3 h) and IFN-γ (3 and 16 h) levels were significantly lower in S1Tg mice than those in WT mice (Fig 2C and 2D). These results suggest that STAP-1 in iNKT cells may be an important adaptor protein that modifies α-GalCer-induced hepatic injury.

### iNKT cells in immune tissues of S1KO and S1Tg mice

**Fig 3:**
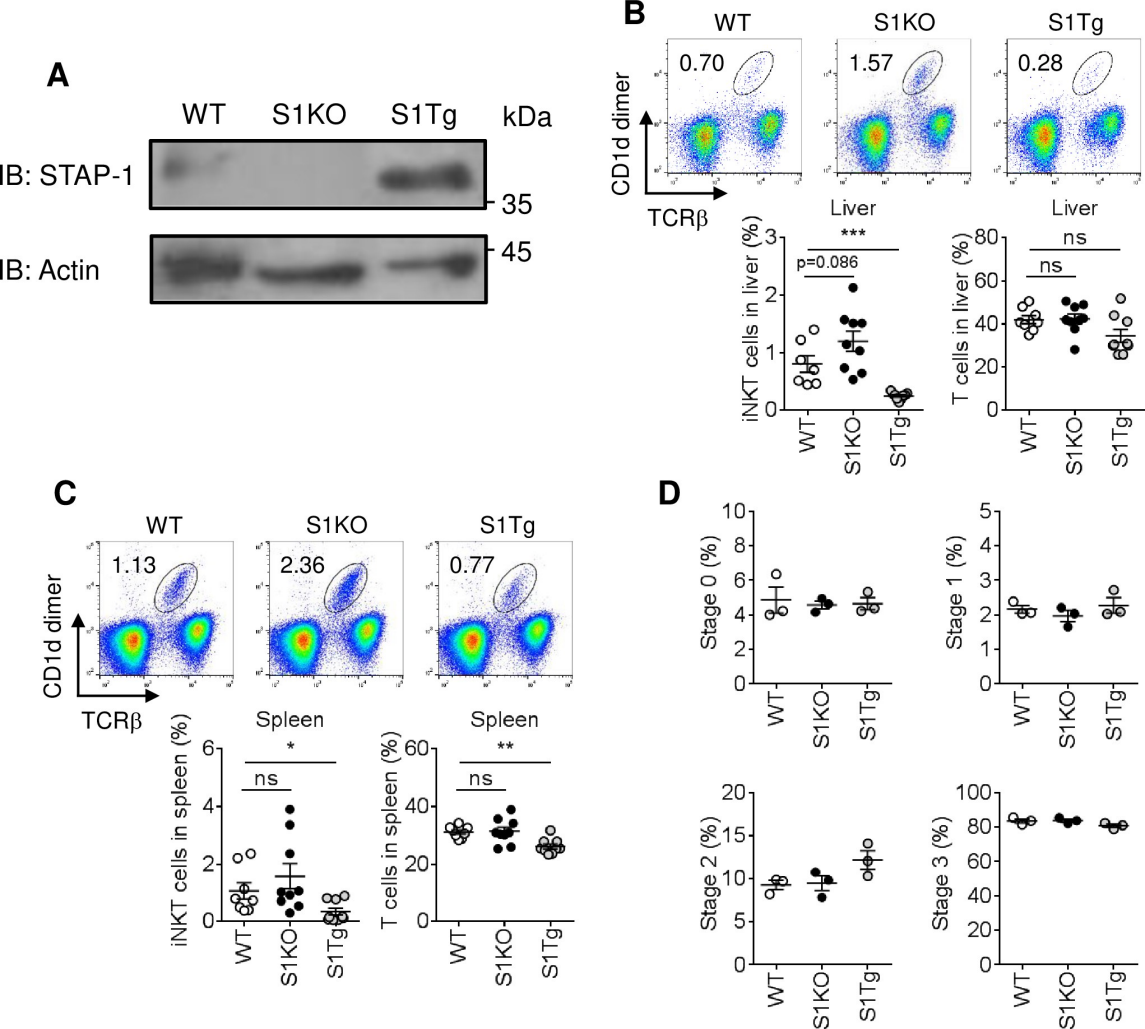
iNKT cell population in STAP-1 KO and Tg mice. **(A)** iNKT cells were purified from spleen and STAP-1 expression was analyzed by western blotting. Actin was detected as a loading control. **(B)** iNKT cell (CD1d dimer^+^/TCRβ^+^) and T cell (CD1d dimer^-^/TCRβ^+^) populations in livers of C57BL/6 (WT), S1KO and S1Tg mice were analyzed by flowcytometric analysis. Upper panels show the representative data of flowcytometric analysis. Lower graphs are summarized data of the flowcytometric analysis. Three independent experiments were performed and pooled data were shown mean + SEM (WT n = 7–8, S1KO n = 9, S1Tg n = 9). **(C)** iNKT cell (CD1d dimer^+^/TCRβ^+^) and T cell (CD1d dimer^-^/TCRβ^+^) populations in spleens of WT, S1KO and S1Tg mice were analyzed by flowcytometric analysis. Upper panels show the representative data of flowcytometric analysis. Lower graphs are summarized data of the flowcytometric analysis. Three independent experiments were performed and pooled data were shown mean + SEM (WT n = 7–8, S1KO n = 9, S1Tg n = 9). **(D)** Analysis of iNKT cell development in thymuses of WT, S1KO and S1Tg mice was performed by flowcytometric analysis. Four figures are summarized graphs of iNKT cell stage 0 to 3. Data were shown mean + SEM of single experiment (WT n = 3, S1KO n = 3, S1Tg n = 3). *; p<0.05, **; p<0.01, ***; p<0.001 by Mann-Whitney U-test. ns = no significance.

First, we analyzed expression of STAP-1 in iNKT cells of WT, S1KO, and S1Tg mice. We observed expression of STAP-1 in WT iNKT cells but not S1KO iNKT cells. We also found enhanced expression levels of STAP-1 was detected in S1Tg iNKT cell compared with WT iNKT cells (Fig 3A). Next, iNKT cell numbers were investigated in the livers and spleens of WT, S1KO, and S1Tg mice. Compared to WT mice, in both the liver and spleen, CD1d dimer^+^/T cell receptor (TCR)β^+^ iNKT cells tended to be increased in S1KO mice, but they were significantly reduced in S1Tg mice (Fig 3B and 3C). However, CD1d dimer^-^/TCRβ^+^ T cell populations were comparable in all three mouse strains with the exception of splenic T cells in S1Tg mice. These results suggest that STAP-1 may be an important adaptor protein in the control of iNKT cell numbers in peripheral tissues. In thymus, based on the expression of CD24, CD44, and NK1.1, CD1d dimer^+^/TCRβ^+^ iNKT cells are further divided according to their differentiation state. There were no significant differences in the proportions of CD24^+^, CD24^-^ NK1.1^-^ CD44^-^, CD24^-^ NK1.1^-^ CD44^+^, or CD24^-^ NK1.1^+^ CD44^+^ populations in the three mouse strains (Fig 3D). These results suggest that STAP-1 may negatively regulate the homeostasis of iNKT cells in peripheral tissues, without influencing early iNKT cell development in the thymus.

### Cytokine production in STAP-1 overexpressing 2E10 iNKT cells after Con A stimulation

**Fig 4:**
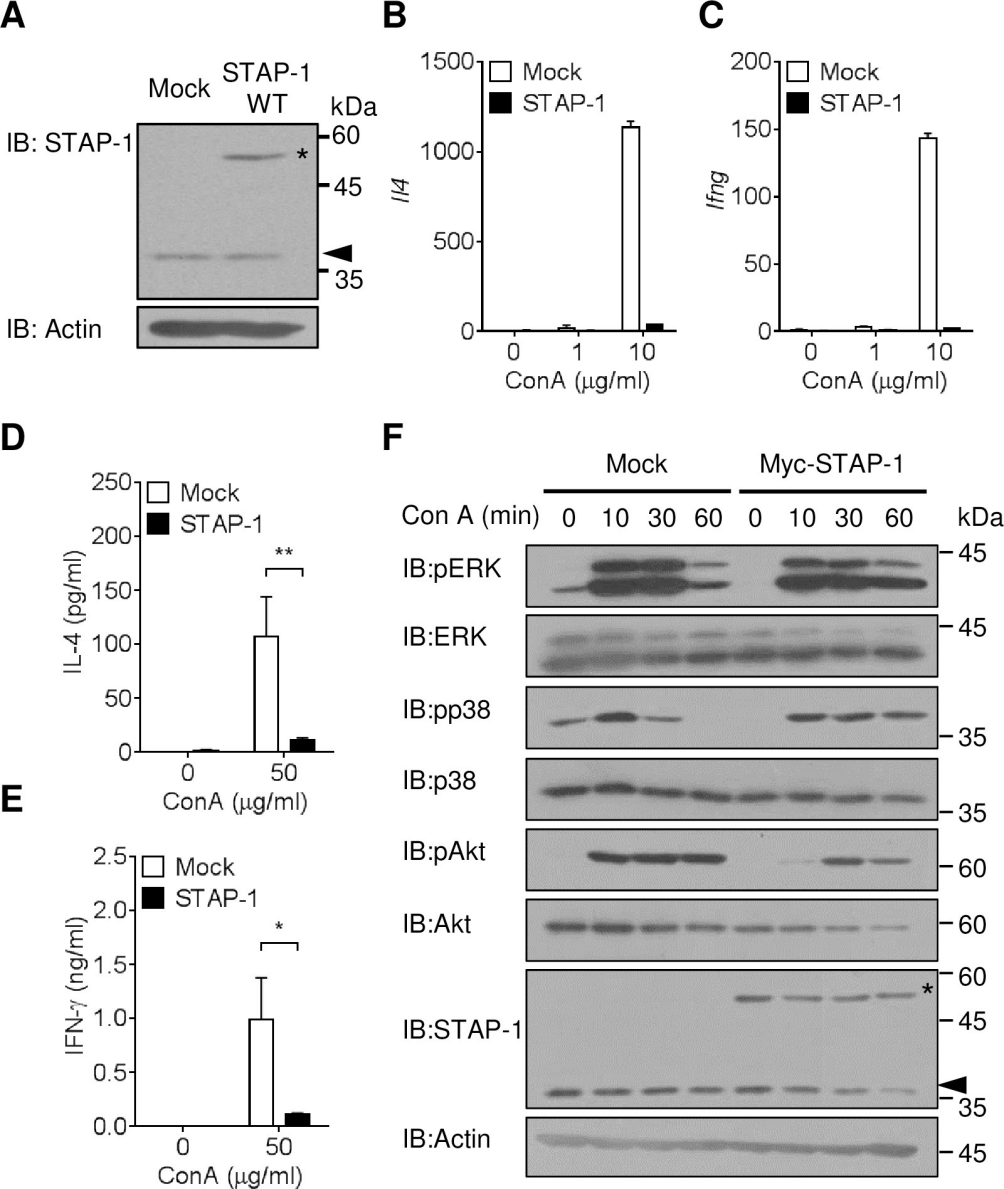
Reduction of cytokine production in STAP-1 2E10 cells. **(A)** Expression of endogenous and exogenous STAP-1 in Mock and STAP-1 2E10 cells was detected by western blotting. Arrowhead and asterisk are shown endogenous and exogenous STAP-1, respectively. Actin was detected as a loading control. **(B, C)** Mock and STAP-1 2E10 cells were stimulated with Con A (0, 1 and 10 μg/ml) for 3 h, and IL-4 and IFN-γ mRNA levels were measured by qPCR. Data were normalized by a ΔΔCt method using GAPDH expression levels. Result shown was representative of two independent experiments. Data was shown mean + SEM. **(D, E)** Mock and STAP-1 2E10 cells were stimulated with Con A (0 and 50 μg/ml) for 24 h, and IL-4 and IFN-γ protein levels in supernatants were measured by ELISA. Data were pooled from 3 independent experiments and shown mean + SEM. *; p<0.05, **; p<0.01 by Mann-Whitney U-test. **(F)** Mock and STAP-1 2E10 cells were stimulated with Con A (10 μg/ml) for indicated times, and phosphorylation of Akt and MAPKs was analyzed by western blotting. Data shown are representative of three independent experiments. Arrowhead and asterisk are shown endogenous and exogenous STAP-1, respectively. Actin was detected as a loading control.

To further clarify the functional roles of STAP-1 in iNKT cell activation, STAP-1 overexpressing 2E10 cells (STAP-1 2E10 cells) were generated. Endogenous STAP-1 (~37 kDa) was expressed in Mock vector-transfected 2E10 cells (Mock 2E10 cells) (Fig 4A). STAP-1 2E10 cells expressed both endogenous and exogenous (~50 kDa) STAP-1. To investigate the effects of STAP-1 on cytokine production from activated iNKT cells, STAP-1 2E10 cells were stimulated with Con A and then IL-4 and IFN-γ mRNA expression levels were measured. When Mock 2E10 cells were stimulated with Con A, levels of IL-4 mRNA expression and IFN-γ mRNA expression were increased. The mRNA expression was dramatically reduced in Con A-stimulated STAP-1 2E10 cells compared with Mock 2E10 cells (Fig 4B and 4C). IL-4 and IFN-γ protein levels were also measured in supernatant of Con A-stimulated Mock and STAP-1 2E10 cell cultures. Concordant with the results of mRNA expression analyses, IL-4 and IFN-γ production was significantly lower in Con A-stimulated STAP-1 2E10 cells than that observed in Mock 2E10 cells (Fig 4D and 4E). Because STAP-1 2E10 cells inhibited Con A-induced IL-4 and IFN-γ production, signal transduction in Mock and STAP-1 2E10 cells was investigated. Upon stimulation with Con A, Mock 2E10 cells exhibited increased phosphorylation of Akt, Erk, and p38. The Con A-induced phosphorylation of Akt and mitogen-activated protein kinases was impaired in STAP-1 2E10 cells (Fig 4F). These results suggest that STAP-1 may be involved in the regulation of Con A-induced signals for iNKT cell activation.

## Discussion

In summary, STAP-1 regulates Con A-induced and/or α-GalCer-induced hepatitis in which iNKT cells have important roles. STAP-1 is involved in at least two events during the pathological process. One is iNKT cell homeostasis in the liver and spleen, and the other is the inhibition of Con A-mediated signals, which leads to reduced IL-4 and/or IFN-γ production. Present data suggest new functions of STAP-1 *in vivo and in vitro*; in addition, STAP-1 is likely to be a key player in the pathogenesis of autoimmune hepatitis.

Con A-induced hepatitis is an experimental model for autoimmune hepatitis, and iNKT cells are required for the development of the type of hepatitis in mice [8]. In this model, hepatocyte apoptosis is regulated by iNKT cells, and Fas/FasL interaction is necessary for iNKT cell-induced hepatocyte apoptosis [31]. Taniguchi and colleagues further demonstrated that iNKT cell-derived IL-4 is crucial for induction of FasL expression in iNKT cells [9]. In the present study, we have shown that S1KO mice produced higher levels of IL-4 and IFN-γ in response to Con A than WT mice, whereas the production of those cytokines was lower in S1Tg mice. Lower IL-4 production by iNKT cells overexpressing STAP-1 may lead to impaired induction of FasL expression. Thus, we speculate that FasL-mediated hepatocyte apoptosis did not occur in S1Tg mice after Con A treatment. Conversely, in S1KO mice, iNKT cells exhibited increased IL-4 production after Con A administration, and higher levels of IL-4 may enhance FasL expression in iNKT cells, resulting in increased apoptosis of hepatocytes. Thus, manipulation of STAP-1 may control iNKT cell activation and autoimmune hepatitis development *in vivo*. One report suggests that leukocyte cell-derived chemotaxin 2 (LECT2) is an important cytokine in the regulation of iNKT cell homeostasis in the liver. LECT2-deficient mice are known to exhibit a reduced number of iNKT cells in the liver, but not the spleen [32]. The differentially targeted organ may indicate that STAP-1 manipulation has effects beyond the modification of LECT2 expression.

CD137, also known as 4-1BB, is a member of the tumor necrosis factor receptor superfamily (TNFRSF) and is expressed on immune cells such as T cells and NK cells. iNKT cells also express CD137, and CD137 signaling is crucial for maintaining iNKT cell numbers in the liver and spleen [33]. Our preliminary study indicated that granzyme B expression in the liver is regulated by STAP-1. In addition, we previously reported that STAP-2 is involved in T cell apoptosis via interaction with another TNFRSF member, Fas (CD95) [34]. Collectively these observations are likely to suggest that STAP-1 may be involved in TNFRSF signaling pathways that include CD137.

Data derived from 2E10 cells overexpressing STAP-1 indicated direct inhibition of signals after Con A stimulation. Although details pertaining to the mechanisms involved remain to be determined, ITK may be a candidate to be regulated by STAP-1 in iNKT cells. ITK is a member of Tec family of tyrosine kinases, and our previous research indicates that STAP-1 binds to another Tec family member, Tec [18]. ITK is known to be expressed by iNKT cells, and it is required for both IFN-γ and IL-4 production in iNKT cells [35]. Our preliminary studies also suggest that ITK may be involved in STAP-1-mediated regulation of T cell activation (unpublished data). We have been reported that STAP family members, especially STAP-2, interact with various signaling molecules such as STAT3, STAT5, MyD88, IKK, PLC-γ1, and caspase-8 [15–17]. Additional detailed research will clarify the molecular mechanisms involved in STAP-1-mediated regulation of signal transduction in iNKT cells, and will provide insights into the development of a novel therapeutic strategy for autoimmune hepatitis.

## Supporting information

S1 File The original uncropped and unadjusted Western blot data. (PDF)

S1 Appendix All raw data presented in this manuscript. (DOCX)
