# Correction: Expression of signal-transducing adaptor protein-1 attenuates experimental autoimmune hepatitis via down-regulating activation and homeostasis of invariant natural killer T cells

**DOI:** 10.1371/journal.pone.0250536

**Published:** 2021-04-15

**Authors:** Jun-ichi Kashiwakura, Kodai Saitoh, Takeru Ihara, Yuto Sasaki, Kota Kagohashi, Shiyo Enohara, Yuka Morioka, Hiroshi Watarai, Ryuta Muromoto, Yuichi Kitai, Kazuya Iwabuchi, Kenji Oritani, Tadashi Matsuda

The following information is missing from the Funding statement: This work was supported in part by JSPS KAKENHI Grants 19H03364 (T.M.) & 16K09872 (K.O.).
