# Supplementary material for: Expression of signal-transducing adaptor protein-1 attenuates experimental autoimmune hepatitis via down-regulating activation and homeostasis of invariant natural killer T cells
Source: PLoS One. 2020 Nov 11;15(11):e0241440. doi: 10.1371/journal.pone.0241440 (PMC7657518; doi:10.1371/journal.pone.0241440)

Fig. 1A Original

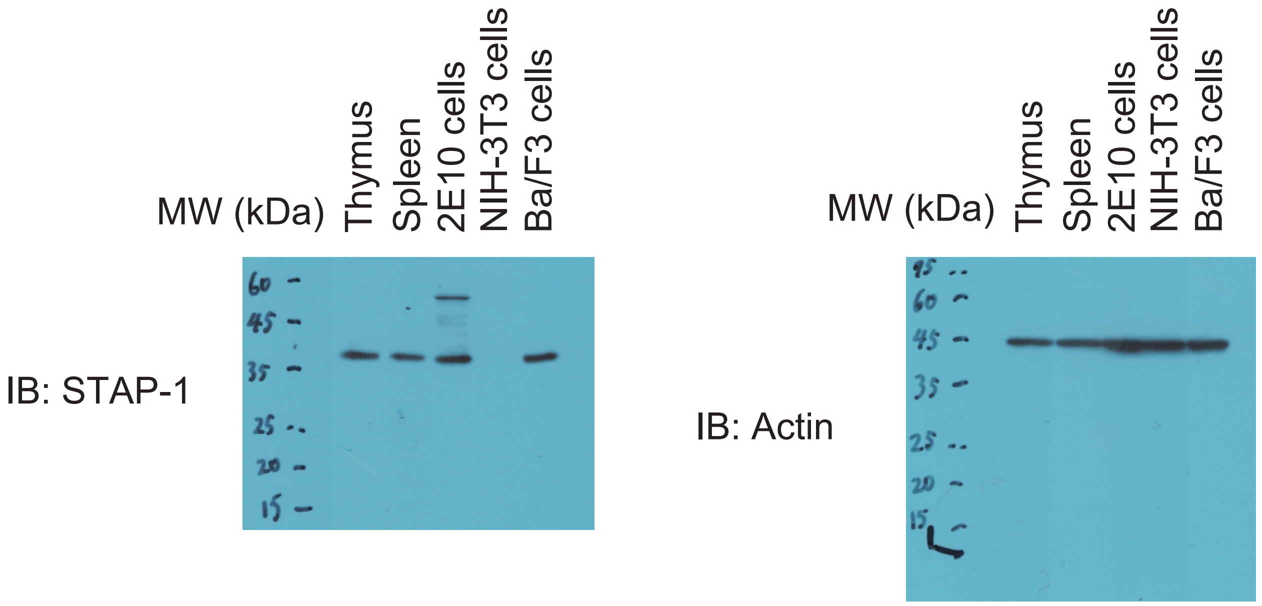

Fig. 1B Original

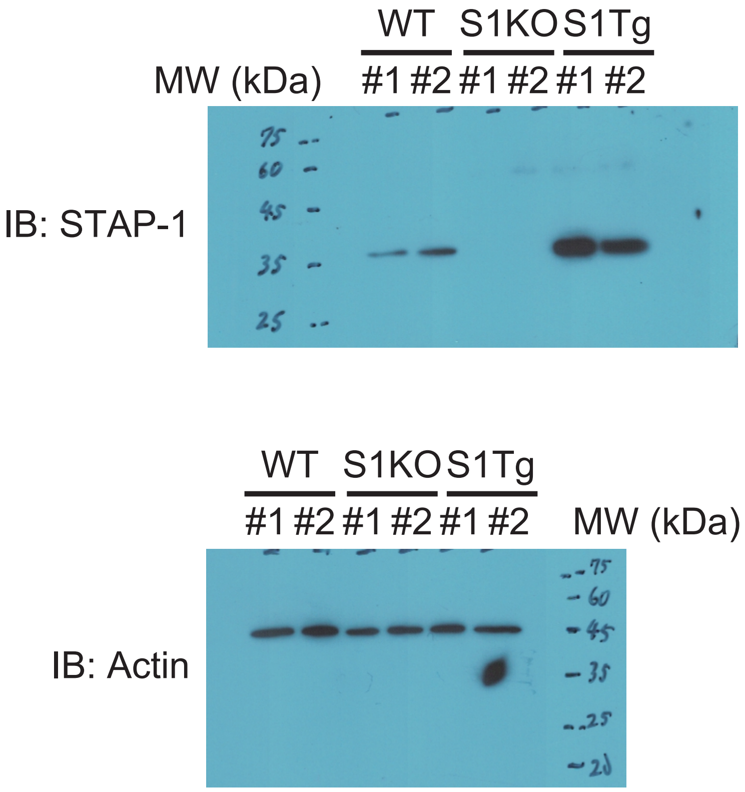

Fig. 1E Original

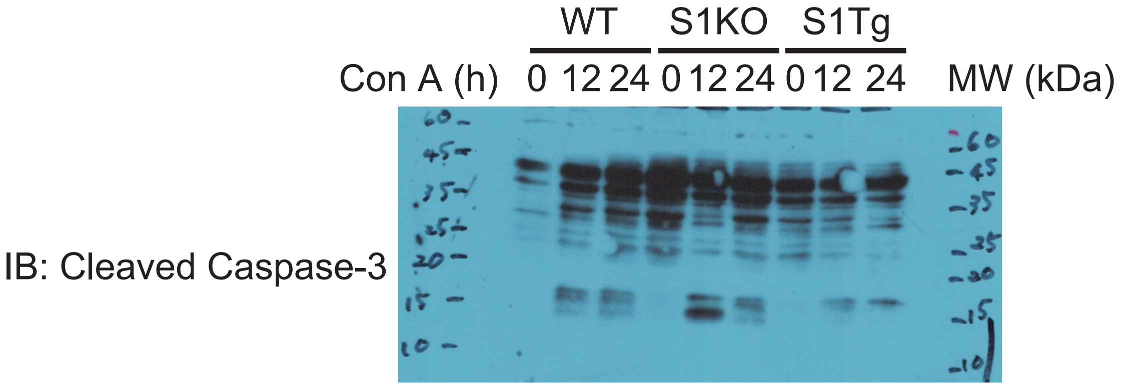

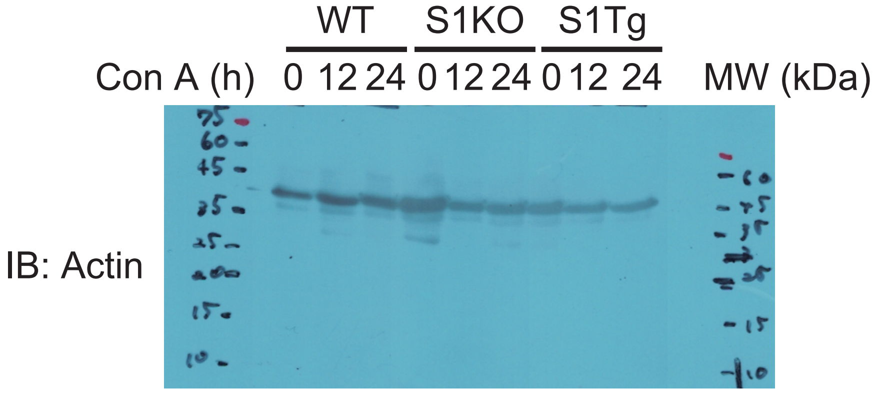

Fig. 3A Original

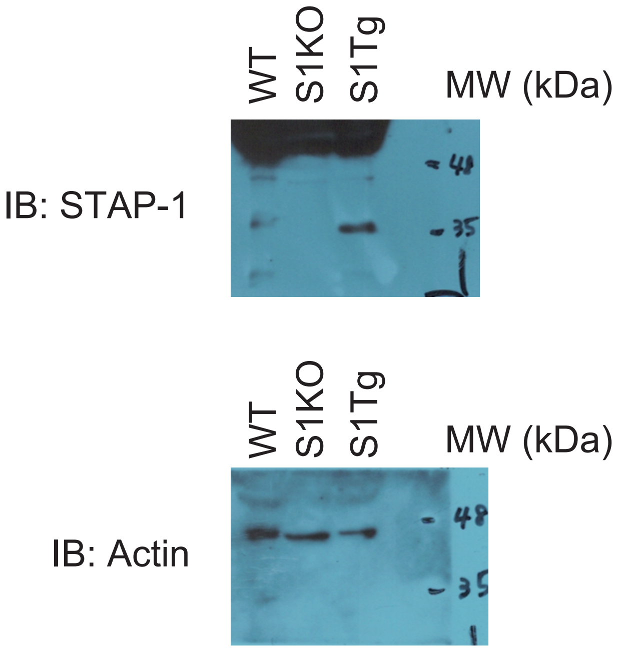

Fig. 4A Original

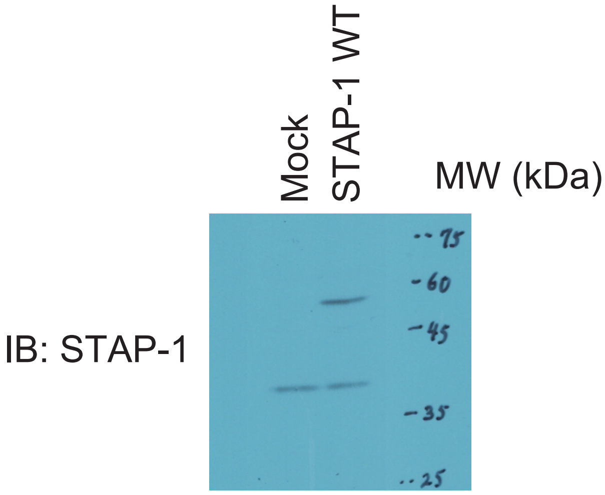

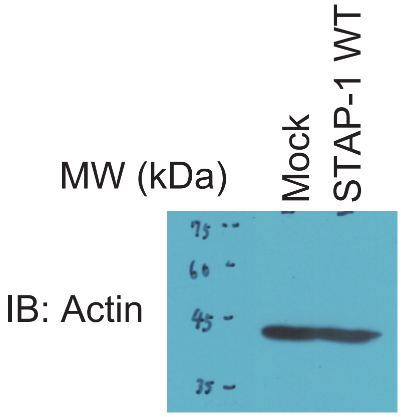

Fig. 4F Original-1

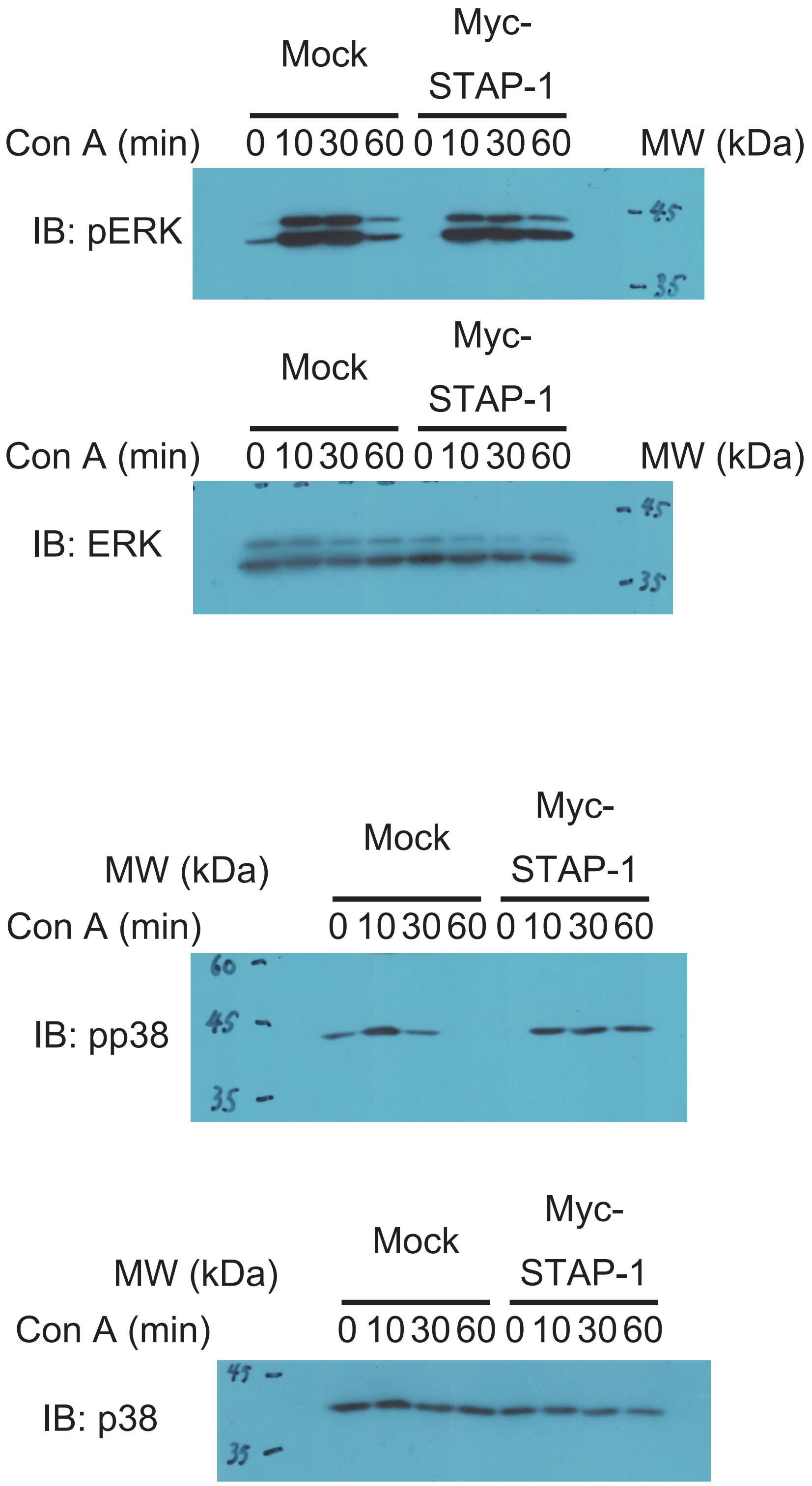

Fig. 4F Original-2

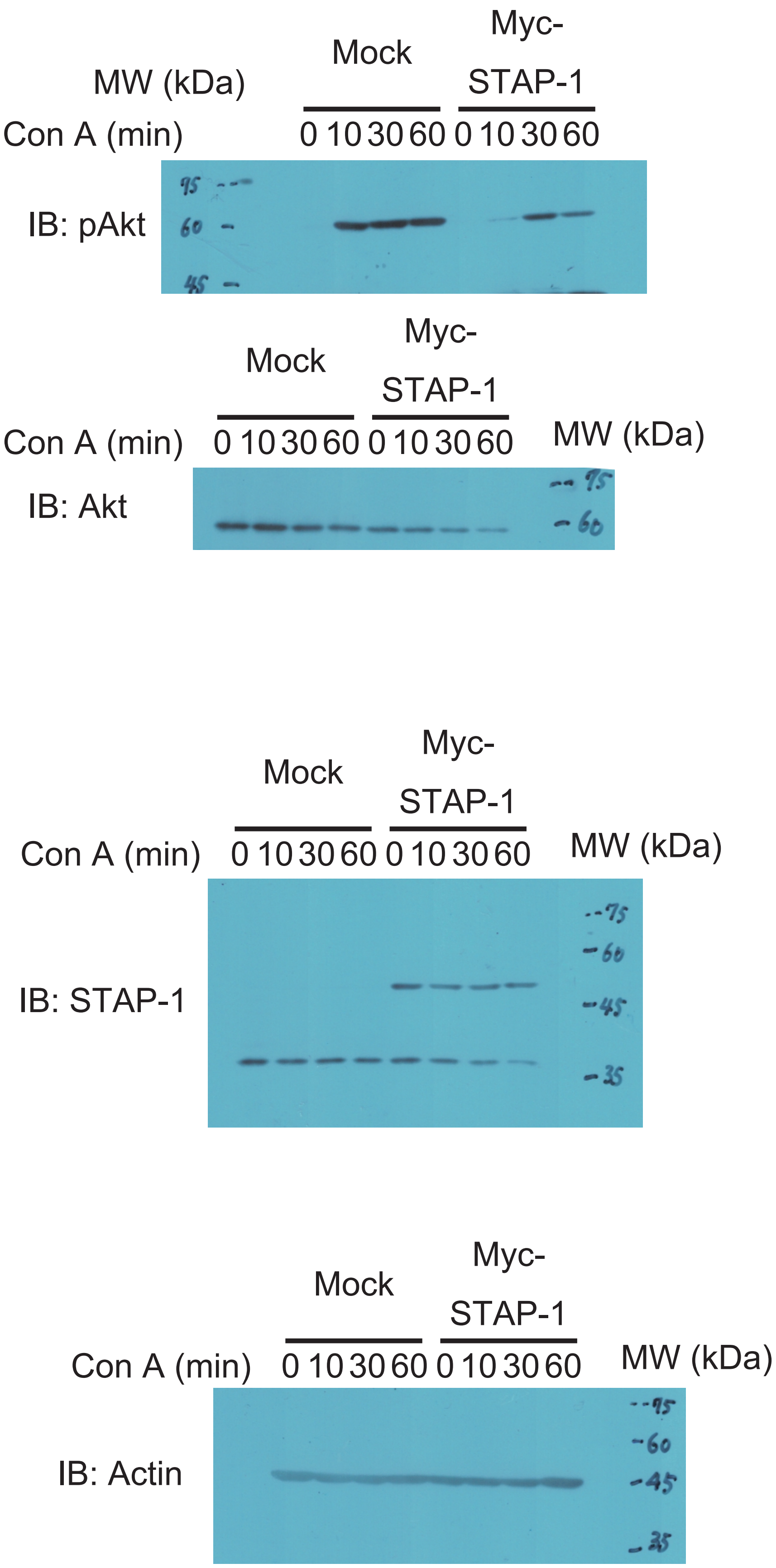

Supplement: S1 File — (PDF) [file pone.0241440.s001.pdf]
