# Supplementary material for: Expression of signal-transducing adaptor protein-1 attenuates experimental autoimmune hepatitis via down-regulating activation and homeostasis of invariant natural killer T cells
Source: PLoS One. 2020 Nov 11;15(11):e0241440. doi: 10.1371/journal.pone.0241440 (PMC7657518; doi:10.1371/journal.pone.0241440)
Supplement: S1 Appendix — (DOCX) [file pone.0241440.s002.docx]

Figure 1D Necrotic area (%) in live of Con A-injected mice

| WT | KO | Tg |
| --- | --- | --- |
| 48.305600 | 60.794900 | 13.260100 |
| 47.454800 | 64.279400 | 13.420500 |
| 42.378800 | 54.637000 | 13.573000 |
| 34.200000 | 58.400000 | 20.100000 |
| 29.800000 | 60.500000 | 17.400000 |
| 32.200000 | 49.700000 | 16.000000 |
| 33.500000 | 41.433330 | 0.000000 |
| 36.833330 | 55.800000 | 10.633330 |
| 37.433330 | 54.966670 | 6.500000 |
| 34.633330 | 61.333330 | 28.100000 |
| 30.700000 | 73.600000 | 23.066670 |
| 51.866660 | 62.333330 | 34.700000 |
| 30.300000 | 77.033330 |  |
| 35.833330 |  |  |

Figure 1F ALT levels (IU/L) in plasma of Con A-injected mice

| WT | KO | Tg |
| --- | --- | --- |
| 2916 | 6726 | 30 |
| 4290 | 6486 | 42 |
| 2154 | 4500 | 30 |
| 2304 | 215 | 45 |
| 2715 | 565 | 70 |
| 2180 | 1835 | 80 |
| 5905 | 3735 | 55 |
| 330 | 15850 | 45 |
| 660 | 1300 | 365 |
| 525 | 5205 | 320 |
| 705 | 13550 | 35 |
| 625 | 13550 | 30 |
| 190 | 1850 |  |
| 120 | 275 |  |
| 110 |  |  |

Figure 1G IL-4 levels (pg/ml) in plasma of Con A-injected mice

| 3h | | | 12h | | |
| --- | --- | --- | --- | --- | --- |
| WT | KO | Tg | WT | KO | Tg |
| 60.894630 | 99.296690 | 18.666580 | 8.291044 | 11.873350 | 0.000000 |
| 40.533840 | 288.435300 | 0.000000 | 15.319040 | 49.391360 | 0.000000 |
| 43.511750 | 74.892800 | 0.000000 | 31.416630 | 11.873350 | 0.000000 |
| 25.687770 | 51.590930 | 8.640600 | 0.000000 | 0.000000 | 0.000000 |
| 40.748310 | 56.299420 | 3.427888 | 0.000000 | 0.000000 | 0.000000 |
| 72.222260 | 95.008810 | 4.691950 | 0.000000 | 0.000000 | 0.000000 |
| 40.748310 | 101.609600 | 5.874282 | 0.000000 | 0.000000 | 0.000000 |
| 64.219530 | 118.217000 | 6.573476 | 0.000000 | 0.000000 | 0.000000 |
| 31.049170 | 171.516100 | 5.874282 | 0.000000 | 0.000000 | 0.000000 |
| 27.007020 | 128.199000 |  | 0.000000 | 0.000000 |  |
| 27.007020 |  |  | 0.000000 |  |  |

Figure 1H IFN-γ levels (ng/ml) in plasma of Con A-injected mice

| 3h | | | 12h | | |
| --- | --- | --- | --- | --- | --- |
| WT | KO | Tg | WT | KO | Tg |
| 0.0913907 | 0.5752075 | 0.1001463 | 1.663463 | 0.972467 | 0.020064 |
| 0.230912 | 0.6081965 | 0.4351165 | 2.886669 | 2.677184 | 0.036299 |
| 0.0574949 | 0.230912 | 0.018739 | 2.339460 | 1.807708 | 0.000000 |
| 0.143821 | 0.194809 | 0.231884 | 1.649840 | 0.671912 | 0.366248 |
| 0.176269 | 0.375513 | 0.227250 | 0.537622 | 1.518956 | 0.078125 |
| 0.398674 | 0.750624 | 0.139185 | 0.375513 | 0.940433 | 0.078125 |
| 0.125277 | 0.570039 | 0.036417 | 0.329186 | 1.495819 | 0.142960 |
| 0.329186 | 0.113117 | 0.061802 | 0.324554 | 2.418132 | 0.211855 |
| 0.040615 | 0.094851 | 0.029325 | 0.468152 | 2.595168 | 0.091043 |
| 0.054905 | 0.059483 |  | 0.934096 | 3.397952 |  |
| 0.044910 |  |  | 1.684984 |  |  |
|  |  |  | 1.626206 |  |  |

Figure 2A Necrotic area (%) in live of α-GalCer-injected mice

| WT | KO | Tg |
| --- | --- | --- |
| 6.605738 | 19.559850 | 2.424963 |
| 13.097090 | 14.618410 | 6.256863 |
| 11.438510 | 22.019130 | 4.009197 |
| 8.864843 | 15.327600 | 2.625137 |
| 10.333330 | 10.333330 | 7.933333 |
| 13.133330 | 13.133330 | 8.000000 |
| 9.150000 | 9.150000 | 11.600000 |
| 9.400000 | 9.400000 | 11.366670 |
| 22.666670 | 22.666670 | 9.800000 |
| 17.366670 | 17.366670 | 10.900000 |
| 18.633330 | 18.633330 |  |

Figure 2B ALT levels (IU/L) in plasma of α-GalCer-injected mice

| WT | KO | Tg |
| --- | --- | --- |
| 218 | 230 | 64 |
| 116 | 178 | 76 |
| 102 | 162 | 80 |
| 140 | 288 | 108 |
| 158 | 224 | 66 |
| 62 | 140 | 140 |
| 55 | 160 | 125 |
| 205 | 225 | 90 |
| 250 | 245 | 145 |
| 245 | 90 | 40 |
| 50 | 120 | 170 |
| 50 | 215 |  |
| 25 | 70 |  |

Figure 2C IL-4 levels (ng/ml) in plasma of α-GalCer-injected mice

| 3h | | | 16h | | |
| --- | --- | --- | --- | --- | --- |
| WT | KO | Tg | WT | KO | Tg |
| 1.295417 | 2.973712 | 0.822297 | 0.000000 | 0.000000 | 0.000000 |
| 1.946854 | 7.401698 | 0.232547 | 0.000000 | 0.000000 | 0.000000 |
| 1.900087 | 8.656453 | 0.117252 | 0.000000 | 0.000000 | 0.000000 |
| 3.647986 | 4.839392 | 0.354633 | 0.000000 | 0.000000 | 0.000000 |
| 3.348689 | 1.838262 | 0.406658 | 0.000000 | 0.000000 | 0.000000 |
| 2.846563 | 1.238334 | 0.331327 | 0.000000 | 0.000000 | 0.000000 |
| 1.234673 | 1.525565 | 0.173853 | 0.000000 | 0.000000 | 0.000000 |
| 1.422080 | 2.135064 | 0.164012 | 0.000000 | 0.000000 | 0.000000 |
| 1.274970 | 1.573744 | 0.210188 | 0.000000 | 0.000000 | 0.000000 |
| 1.374173 | 1.707583 | 0.206870 | 0.000000 | 0.000000 | 0.000000 |
| 0.940327 | 1.644307 | 0.430797 | 0.000000 | 0.000000 | 0.000000 |
| 1.088767 | 2.597837 |  | 0.000000 | 0.000000 |  |
| 1.388904 | 1.355771 |  | 0.000000 | 0.000000 |  |
| 1.176190 |  |  | 0.000000 |  |  |

Figure 2D IFN-γ levels (ng/ml) in plasma of α-GalCer-injected mice

| 3h | | | 16h | | |
| --- | --- | --- | --- | --- | --- |
| WT | KO | Tg | WT | KO | Tg |
| 7.085856 | 11.76105 | 0.23197 | 0.14322 | 0.754365 | 0.051916 |
| 4.930095 | 14.04613 | 0.476675 | 0.454878 | 0.928368 | 0.065646 |
| 8.850375 | 16.41751 | 0.289392 | 0.110622 | 0.476675 | 0.015965 |
| 4.419792 | 20.42446 | 0.520937 | 0.611989 | 2.296468 | 0.110622 |
| 0.737101 | 22.83521 | 0.754365 | 0.754365 | 14.94768 | 0.489722 |
| 2.802074 | 6.643007 | 1.39845 | 0.476675 | 41.60583 | 0.700062 |
| 2.236959 | 1.706071 | 0.86723 | 8.334832 | 7.842383 | 0.591848 |
| 2.152951 | 1.002519 | 0.161912 | 7.92378 | 14.84757 | 0.814017 |
| 0.552555 | 3.211415 | 0.100408 | 7.599841 | 36.13835 |  |
| 4.021324 | 1.627725 | 0.032271 | 12.04189 | 4.278374 |  |
| 1.436045 | 0.86723 | 0.161912 | 5.678349 | 16.47524 |  |
| 0.86723 | 1.071985 | 0.612564 | 1.751807 | 30.67414 |  |
|  | 4.468106 |  |  | 16.06318 |  |
|  | 1.287205 |  |  |  |  |

Figure 3A Population of iNKT cells and T cells (%) in liver

| iNKT cells | | | T cells | | |
| --- | --- | --- | --- | --- | --- |
| WT | KO | Tg | WT | KO | Tg |
| 0.70 | 2.13 | 0.28 | 34.80 | 37.70 | 25.70 |
| 1.22 | 1.57 | 0.34 | 40.70 | 28.10 | 30.00 |
| 0.87 | 1.51 | 0.29 | 37.10 | 41.70 | 29.90 |
| 0.44 | 0.64 | 0.19 | 39.50 | 50.50 | 30.40 |
| 0.46 | 0.53 | 0.26 | 50.50 | 47.80 | 30.80 |
| 0.51 | 1.03 | 0.26 | 48.00 | 48.90 | 25.80 |
| 1.40 | 1.51 | 0.25 | 40.60 | 41.80 | 43.90 |
|  | 1.14 | 0.13 | 44.10 | 42.50 | 51.70 |
|  | 0.73 | 0.20 |  | 41.10 | 41.10 |

Figure 3B Population of iNKT cells and T cells (%) in spleen

| iNKT cells | | | T cells | | |
| --- | --- | --- | --- | --- | --- |
| WT | KO | Tg | WT | KO | Tg |
| 1.130 | 3.900 | 0.890 | 32.600 | 25.300 | 25.400 |
| 2.200 | 3.360 | 0.810 | 28.800 | 25.900 | 25.400 |
| 2.360 | 2.360 | 0.770 | 28.300 | 30.900 | 27.800 |
| 0.400 | 0.710 | 0.067 | 30.600 | 34.100 | 31.700 |
| 0.490 | 0.530 | 0.051 | 30.000 | 35.700 | 25.300 |
| 0.380 | 0.290 | 0.046 | 31.700 | 38.900 | 24.700 |
| 0.790 | 1.060 | 0.150 | 34.200 | 30.900 | 23.600 |
| 0.730 | 1.020 | 0.120 | 32.700 | 30.100 | 23.300 |
|  | 0.920 | 0.170 |  | 30.400 | 27.800 |

Figure 3C Population of each iNKT stage (%) in thymus

| Stage 0 | | | Stage 1 | | |
| --- | --- | --- | --- | --- | --- |
| WT | KO | Tg | WT | KO | Tg |
| 4.01000 | 4.15000 | 5.33000 | 2.06400 | 2.03308 | 2.08340 |
| 4.22000 | 4.64000 | 4.37000 | 2.04054 | 1.65042 | 2.02672 |
| 6.36000 | 4.94000 | 4.22000 | 2.38680 | 2.20632 | 2.72072 |

| Stage 2 | | | Stage 3 | | |
| --- | --- | --- | --- | --- | --- |
| WT | KO | Tg | WT | KO | Tg |
| 9.888000 | 7.835030 | 10.322300 | 83.616000 | 85.351000 | 82.294300 |
| 8.200481 | 9.826200 | 12.045600 | 85.453600 | 83.665800 | 80.973200 |
| 9.734400 | 10.746300 | 14.082600 | 81.432000 | 81.976200 | 78.747600 |

Figure 4B IL-4 mRNA expression levels in activated 2E10

|  | IL-4 | | | |
| --- | --- | --- | --- | --- |
| ConA (μg/ml) | Mock | | STAP-1 | |
| 0 | 1.741173 | 0.2588269 | 0.1866793 | 1.813321 |
| 1 | 8.694238 | 34.536730 | 2.905193 | 4.108563 |
| 10 | 1105.175000 | 1168.190000 | 146.894200 | 139.937000 |

Figure 4C IFN-γ mRNA expression levels in activated 2E10

|  | IL-4 | | | |
| --- | --- | --- | --- | --- |
| ConA (μg/ml) | Mock | | STAP-1 | |
| 0 | 0.360641 | 0.499527 | 0.360641 | 0.499527 |
| 1 | 1.041479 | 1.327428 | 1.041479 | 1.327428 |
| 10 | 2.040092 | 2.600221 | 2.040092 | 2.600221 |

Figure 4D IL-4 levels (pg/ml) in supernatants of activated 2E10

| Mock | | STAP-1 | |
| --- | --- | --- | --- |
| Con A  0 μg/ml | Con A  50 μg/ml | Con A  0 μg/ml | Con A  50 μg/ml |
| 0.000000 | 243.418700 | 6.172227 | 20.143890 |
| 0.133969 | 91.743700 | 0.087420 | 4.133339 |
| 0.615596 | 192.703400 | 2.860633 | 9.413840 |
| 0.000000 | 24.038370 | 0.000000 | 10.812450 |
| 0.000000 | 36.937300 | 0.000000 | 14.598500 |
| 0.000000 | 55.915200 | 0.000000 | 9.216150 |

Figure 4E IFN-γ levels (ng/ml) in supernatants of activated 2E10

| Mock | | STAP-1 | |
| --- | --- | --- | --- |
| Con A  0 μg/ml | Con A  50 μg/ml | Con A  0 μg/ml | Con A  50 μg/ml |
| 0.000000 | 0.187451 | 0.000000 | 0.107736 |
| 0.000000 | 0.119334 | 0.000000 | 0.089057 |
| 0.000000 | 1.434731 | 0.000000 | 0.126987 |
| 0.000000 | 2.160000 | 0.000000 | 0.071512 |
| 0.000000 | 1.057990 | 0.000000 | 0.160266 |
